# Effects of Signal Disruption Depends on the Substrate Preference of the Lactonase

**DOI:** 10.3389/fmicb.2019.03003

**Published:** 2020-01-14

**Authors:** Kathleen Mahan, Ryan Martinmaki, Isabel Larus, Rakesh Sikdar, Jordan Dunitz, Mikael Elias

**Affiliations:** ^1^Division of Pulmonary, Allergy, Critical Care and Sleep Medicine, Department of Medicine, University of Minnesota, Minneapolis, MN, United States; ^2^Department of Biochemistry, Molecular Biology and Biophysics, Biotechnology Institute, University of Minnesota, Saint Paul, MN, United States; ^3^Department of Medicine, Minnesota Cystic Fibrosis Center and Adult CF Program, University of Minnesota, Minneapolis, MN, United States

**Keywords:** quorum sensing, lactonase, *Pseudomonas aeruginosa*, cystic fibrosis, quorum quenching, signaling, biofilm

## Abstract

Many bacteria produce and use extracellular signaling molecules such as acyl homoserine lactones (AHLs) to communicate and coordinate behavior in a cell-density dependent manner, *via* a communication system called quorum sensing (QS). This system regulates behaviors including but not limited to virulence and biofilm formation. We focused on *Pseudomonas aeruginosa*, a human opportunistic pathogen that is involved in acute and chronic lung infections and which disproportionately affects people with cystic fibrosis. *P. aeruginosa* infections are becoming increasingly challenging to treat with the spread of antibiotic resistance. Therefore, QS disruption approaches, known as quorum quenching, are appealing due to their potential to control the virulence of resistant strains. Interestingly, *P. aeruginosa* is known to simultaneously utilize two main QS circuits, one based on C4-AHL, the other with 3-oxo-C12-AHL. Here, we evaluated the effects of signal disruption on 39 cystic fibrosis clinical isolates of *P. aeruginosa*, including drug resistant strains. We used two enzymes capable of degrading AHLs, known as lactonases, with distinct substrate preference: one degrading 3-oxo-C12-AHL, the other degrading both C4-AHL and 3-oxo-C12-AHL. Two lactonases were used to determine the effects of signal disruption on the clinical isolates, and to evaluate the importance of the QS circuits by measuring effects on virulence factors (elastase, protease, and pyocyanin) and biofilm formation. Signal disruption results in at least one of these factors being inhibited for most isolates (92%). Virulence factor activity or production were inhibited by up to 100% and biofilm was inhibited by an average of 2.3 fold. Remarkably, the treatments led to distinct inhibition profiles of the isolates; the treatment with the lactonase degrading both signaling molecules resulted in a higher fraction of inhibited isolates (77% vs. 67%), and the simultaneous inhibition of more virulence factors per strain (2 vs. 1.5). This finding suggests that the lactonase AHL preference is key to its inhibitory spectrum and is an essential parameter to improve quorum quenching strategies.

## Introduction

Quorum sensing (QS) is the communication system used by many bacteria to coordinate behaviors including those important for causing disease in humans. This process was first discovered in the 1970’s with the observation of the illumination of organs within certain marine species (Nealson et al., 1970). This phenomenon of “bioluminescence” was soon found to be a concentration-dependent process mediated by signaling molecules produced by bacteria as they increased in cell density, termed “autoinduction” by Nealson et al. (1970). Gram negative and Gram positive bacteria use QS to coordinate gene expression for behaviors that are vital to their survival such as denitrification and glucose metabolism, as well as those important for causing disease (Miller and Bassler, 2001; Heurlier et al., 2005; Lee and Zhang, 2015; Mukherjee and Bassler, 2019). This includes the regulation of key factors involved in activities ranging from motility, evasion of the host immune system, production of scavenging molecules, and production of directly cytotoxic molecules (Lee and Zhang, 2015).

This complex system is vital to the pathogenicity of many bacteria toward humans and the ability to decipher, and disrupt the system could provide an alternative or adjunctive treatment approach to antibiotics. In the model organism *Pseudomonas aeruginosa*, an opportunistic pathogen, as much as 12% of the bacterial genome has been identified to be under QS control (Heurlier et al., 2005; Kalia, 2015; Lee and Zhang, 2015). *P. aeruginosa* is known to utilize two QS systems based on acyl homoserine lactones (AHLs) as the signaling molecule (LaSarre and Federle, 2013; Lee and Zhang, 2015). This has been shown in both wild type and clinical isolates (Feltner et al., 2016). These systems are known as *las* and *rhl*, named for their transcription factor regulators, LasR, and RhlR and utilize 3-oxo-dodecanoyl homoserine lactone (3-oxo-C12-HSL), and N-butyryl homoserine lactone (C4-HSL), respectively. The QS systems in *P. aeruginosa*, as in other bacteria, are complex, hierarchical, and adaptable. Studies show that the currently understood hierarchical system can be altered in clinical isolates of *P. aeruginosa* which have lived in human lungs for years (Bjarnsholt et al., 2010; Feltner et al., 2016; Chen et al., 2019; Kostylev et al., 2019). There is redundancy in the transcriptional control of certain gene products but certain virulence factors essential to pathogenicity are under strictly regulated QS control; for example, pyocyanin production is a product of a complex metabolic pathway positively controlled by the transcriptional regulator, RhlR (Nadal Jimenez et al., 2012; Higgins et al., 2018). In addition, some of these strains can lose the ability to respond to QS: they are called social cheaters (Sandoz et al., 2007; Popat et al., 2012; Mukherjee and Bassler, 2019). These strains have reduced pathogenicity and may instead exist in a quiescent manner (Heurlier et al., 2005; Popat et al., 2012). They may also be less fit than QS-responsive strains (Gerdt and Blackwell, 2014) and dependent on non-cheater strains (Köhler et al., 2009; Winstanley and Fothergill, 2009). While QS still appears to be essential in late stages of chronic lung infections (Winstanley and Fothergill, 2009; Bjarnsholt et al., 2010), it is unclear how efficient strategies pertaining to the inhibition of QS would be against *P. aeruginosa* strains that can be subjected to these regulatory alterations.

Inhibition of bacterial QS based on AHLs can be performed using lactonases, enzymes that degrade lactones, including AHLs. Consequently, these enzymes were previously reported to inhibit the behaviors regulated by QS, including biofilm and virulence products during *in vitro* and *in vivo* experiments (Dong et al., 2000; Cao et al., 2012; Hraiech et al., 2014; Vinoj et al., 2014; Gupta et al., 2015; Guendouze et al., 2017; Bergonzi et al., 2018). These enzymes therefore constitute promising candidates to control bacterial virulence and biofilms (Whiteley et al., 2017). Using lactonases may be advantageous to control virulence and biofilm formation over other strategies because these enzymes are not biocidal, and were previously shown to not need contact with bacteria for their activity (Oh et al., 2012; Schwab et al., 2019). Therefore, the risk of resistance (Defoirdt et al., 2010) may be lessened compared to antibiotics (Gerdt and Blackwell, 2014; García-Contreras et al., 2016).

Lactonases are naturally occurring enzymes and can be found in a variety of organisms, including bacteria, archaea, plants, fungi, and mammals (Elias and Tawfik, 2012; LaSarre and Federle, 2013). Lactonases can be found in various protein families, including the paraoxonases (PONs) (Khersonsky and Tawfik, 2005; Ben-David et al., 2012, 2013), the phosphotriesterase-like lactonases (PLLs) (Afriat et al., 2006; Elias and Tawfik, 2012; Hiblot et al., 2013, 2015; Bzdrenga et al., 2014) and the metallo-β-lactamases lactonases (MLLs) (Liu et al., 2007, 2008; Momb et al., 2008; Mascarenhas et al., 2015; Tang et al., 2015; Bergonzi et al., 2016, 2018). Remarkably, while AHLs vary considerably in their chemical structure, and in particular, the nature and length of their acyl chain, recent work on lactonase kinetic properties suggest, in contrast, a low variety in the lactonase’s substrate specificities. In fact, most characterized lactonases exhibit two types of substrate preferences: (i) very broad substrate specificity (e.g., MLLs) (Tang et al., 2015; Bergonzi et al., 2016, 2017, 2018) or (ii) a preference for longer acyl chains (e.g., PLLs and PONs) (Hiblot et al., 2012b, 2013; Bar-Rogovsky et al., 2013; Bzdrenga et al., 2014).

Here, we took advantage of the distinct substrate preference of the PLL, SsoPox (Hiblot et al., 2013), which prefers longer AHL molecules, and the MLL, GcL (Bergonzi et al., 2019), which exhibits very broad substrate specificity. We used these lactonases to study the effects of AHL signal disruption on *P. aeruginosa* clinical isolates from cystic fibrosis (CF) patients. Due to their substrate specificity, these lactonases can be used to selectively disrupt the Las QS circuit (with SsoPox), or both QS circuits, Las and Rhl (with GcL) in *P. aeruginosa.* Therefore, we have investigated (i) the effects of differential signal disruption on the production of virulence factors and biofilm formation and (ii) evaluated the ability of lactonases to inhibit clinical isolates of *P. aeruginosa* with high propensity for remodeling of their QS circuits (D’Argenio et al., 2007; Hoffman et al., 2009; Bjarnsholt et al., 2010).

We found that both lactonases can inhibit virulence factor production and biofilm formation of most clinical isolates of *P. aeruginosa*. This is important because it provides evidence to the potential of quorum quenching enzymes to control pathogens, including drug resistant clinical isolates from CF patients. *P. aeruginosa* is indeed the predominant opportunistic pathogen that causes significant morbidity and mortality in cystic fibrosis patients, as well as in individuals with other acute or chronic lung disease (Hauser et al., 2011; Ciofu et al., 2013), and there is great need for new or adjunctive therapy. Additionally, while there is overlap in the strains that are inhibited by both lactonases (20 strains, over a total of 36 strains inhibited, 55%), there are in fact a significant number of strains that are inhibited by only one lactonase (16 strains, 44% of 36). In addition, the treatment with each lactonase results in a different average of inhibited traits per strain (10 for SsoPox, 15 for GcL). Therefore, this study, as the first evaluation of the importance of the lactonase substrate specificity on clinical isolates, reveals that the preference of the lactonase is key to affecting the number and nature of the inhibited traits for each bacterial isolate.

## Materials and Methods

### Bacterial Strains

Experiments were performed with *P. aeruginosa* strains obtained by the Hunter Lab from the University of MN Department of Microbiology and Immunology and MN Cystic Fibrosis Center. The clinical strains were isolated from patients with cystic fibrosis. All the patients received oral information, were anonymized and were given a non-opposition statement to bacterial storage. This study was approved by the University of Minnesota IRB and was carried out in accordance with the Declaration of Helsinki as revised in 2008. The samples were frozen at −80°C. Bacterial strains were cultivated on Luria Bertani (LB) agar plates at 37°C.

The clinical isolates, WT strain PA14, or mutant strains, LasRΔ, RhlIΔ (SM52), or RhlRΔ (SM32) were inoculated from a single colony and grown at 37°C in Luria Bertani media (LB – 10 g/L NaCl, 10 g/L tryptone, 5 g/L yeast extract) with shaking at 250 rpm until OD600_nm_ of 0.1. Subsequently, 2 mL of LB was inoculated at 1:1000 dilution with pre-culture and incubated at 37°C with shaking at 250 rpm. Protease, elastase, biofilm and pyocyanin production were measured 20 h post-inoculation as described below. SsoPox-W263I was added at a final concentration of 125 μg/mL, GcL was added at a final concentration of 55 μg/mL. The quorum sensing inhibitor (QSI) 5-Fluoro-Uracil (5-FU) was used at 60 μM, and bovine serum albumin (BSA) was added at 100 μg/mL, both final concentrations. See Supplementary Table S3 for a full list of strains.

### Protein Production and Purification

Enzyme production was performed using the Escherichia *coli* BL21 (DE3)-pGro7/GroEL strain (Takara). SsoPox-W263I enzyme was produced and purified as previously described (Hiblot et al., 2012a) and GcL was produced and purified as previously characterized (Bergonzi et al., 2016).

### Proteolytic Activity

Cell-free culture supernatants were prepared by centrifugation for 10 min at 2272*g*. Protease activity was determined using azocasein (Sigma-Aldrich, Burlington, MA, United States) as a substrate. The reaction was performed in 675 μl phosphate-buffered saline (PBS) solution pH 7.0 with 50 μl of azocasein (30 mg/mL in water) and with 25 μl of culture supernatant for a final volume of 750 μl. The reaction was incubated at 37°C for 2 h and stopped by adding 125 μl of 20% (w/v) trichloroacetic acid. A blank was performed with each assay with the substitution of media for lactonase. After centrifugation at 10,000*g* for 10 min, the absorbance of the supernatant was measured at 366 nm using a plate reader (Synergy HTX, BioTek, United States). Experiments were performed in quadruplicate in 96 well-plates.

### Elastase Activity

Cell-free culture supernatants were prepared by centrifugation for 10 min at 2272*g*. Elastase activity was measured using 5 mg/mL elastin congo red (ECR) (Sigma-Aldrich, Burlington, MA, United States) as a substrate in a 10 mM Tris solution. The reaction was performed with 150 μl ECR and 50 μl of culture supernatant for a final volume of 200 μl. A blank was performed with each assay with the substitution of media for lactonase. The reaction was covered with aluminum foil and incubated at 37°C for 24 h with agitation. After resting the plate for 5 min, 100 μl of each reaction was transferred to a new plate and the absorbance of the supernatant was measured at 490 nm using a plate reader (Synergy HTX, BioTek, United States). Experiments were performed in quadruplicate in 96 well-plates.

### Biofilm Quantification

Cell-free culture supernatants were prepared by centrifugation for 10 min at 2272*g*. Culture supernatants were transferred to a new microtiter plate with care to avoid unsettling the formed biofilm. The biofilm was washed with 200 μl sterile water three times and the plate was allowed to dry for 30 min. 200 μl of 0.1% crystal violet was added to each biofilm and allowed to incubate with mild rotation for 30 min. The crystal violet was then washed off and the plate was allowed to dry for 30 min. Subsequently, 200 μl of 30% acetic acid was added to dissolve the stained biofilm. Dissolved biofilm was transferred to a new plate and the OD_550__nm_ was read with a plate reader (Synergy HTX, BioTek, United States). Experiments were performed in quadruplicate in 96 well-plates.

### Pyocyanin Measurement

Cell-free culture supernatants were prepared by centrifugation for 10 min at 2272*g*. Supernatants were transferred to a new microtiter plate and the absorbance of the supernatant was measured at 691 nm using a plate reader (Synergy HTX, BioTek, United States). Experiments were performed in quadruplicate in 96 well-plates. This method was previously described (Das and Manefield, 2012).

### Acyl Homoserine Lactone Quantification

Acyl homoserine lactones quantification was done as previously described (Luong et al., 2014). *E. coli* biosensor strain JM109 pSB536 or S17 pSB1075 (Swift et al., 1997; Winson et al., 1998; Moorhead and Griffiths, 2011) was grown overnight in LB containing 100 μg/mL ampicillin at 37°C and shaking at 250 rpm. Next morning, the overnight culture was diluted 1:50 into fresh LB containing 100 μg/mL ampicillin and grown at 37°C at 250 rpm for 2 h. 190 μL of biosensor cultures were then mixed with 10 μL of cell-free supernatants of stationary phase cultures of *P. aeruginosa* in solid-white non-binding 96-well microplates with clear well bottoms (Grenier Bio-One). For time-course assays, supernatants were obtained from cultures incubated with lactonase after 4 h or 20 h. Additionally, BSA, SsoPox or GcL was added to the aforementioned supernatant mixtures at a final concentration of 100 μg/mL. Blank reactions were set up for all conditions containing 200 μL of biosensor cultures only. C4-HSL or 3OC12-HSL were used as positive controls for JM109 pSB536 or S17 pSB1075 biosensors, respectively, at a final concentration of 300 nM. The microplate was then incubated at 37°C at 300 rpm for 2 h. The luminescence was read at a gain of 180 for an integration time of 10 s per well using a Synergy HTX plate reader (BioTek, United States). The final OD_600_ of the cultures in each well was recorded by the plate reader simultaneously.

### Antibiotic Sensitivity Measurement

A single colony was inoculated in 2 mL LB and incubated with shaking at 250 rpm until OD600_nm_ of 0.5 was reached. Subsequently, a bacterial lawn was spread onto an LB agar plate which contained bovine serum albumin (BSA, Sigma-Aldrich, Burlington, MA, United States) as a control or lactonase (data not reported here). Antibiotic discs (levofloxacin, cefepime, or piperacillin-tazobactam; Thermo Fisher Scientific, Lenexa, KS, United States) were placed in the respective sections of the petri dish and the plates were incubated at 37°C for 48 h. Zone of inhibition (ZOI) was measured to the nearest millimeter (mm) at 24 h (not shown) and 48 h. ZOI standards for the respective antibiotics were evaluated based on Clinical and Laboratory Standards Institute (CLSI).

### Amplification of the *lasr* Gene

DNA obtained through spot colony amplification was performed on all 39 clinical isolates of *P. aeruginosa* using a T100^TM^ Biorad Thermal Cycler (Hercules, CA, United States) as described previously (Bergkessel and Guthrie, 2013). Amplification of the 1.24-kb *lasr* region was performed using the following primers: las1 CGCCGAACTGGAAAAGTGGC, upstream of *lasr*; las2 TGAGAGGCAAGATCAGAGAG, downstream of *lasr*, as previously described (Heurlier et al., 2005). PA14 and PAO1 were used as a positive control and *E. coli* OP50 and PA14 LasRΔ was used as a negative control.

### Statistical Analysis

Student’s one-tailed *t* test was performed on appropriate strains comparing untreated to treated (BSA, 5-FU, 5A8, Ssopox, or GcL) using GraphPad Prism Software (CA, United States). Tests were considered significant for *p* values ≤ 0.05. Symbols were associated with significant *p* values; *p* ≤ 0.05 (^∗^), *p* ≤ 0.01 (^∗∗^), and *p* ≤ 0.001 (^∗∗∗^). Spearman correlation coefficients were calculated using GraphPad Prism Software (CA, United States). Inhibitory levels were calculated as the reduction in the measure of virulence factors or biofilm between control and lactonase treatments. The average inhibitory level is the mean of all inhibition parameters observed for a given measured virulence factor and a given treatment for all clinical isolates. In these calculations, only reductions with statistical significance (*p* ≤ 0.05) were included.

## Results and Discussion

### Both Lactonases Degrade Acyl Homoserine Lactones in Cultures, but They Show Distinct Preferences

**FIGURE 1:**
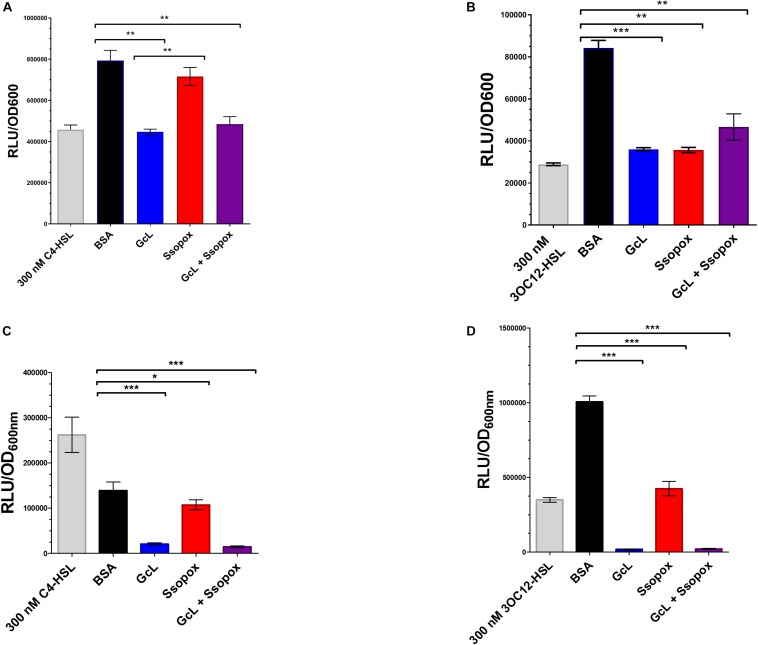
Lactonases target and effectively hydrolyze specific acyl homoserine lactones (AHLs). **(A)** AHL detection using Escherichia *coli* based sensor plasmid, pSB536, which specifically detects C4-homoserine lactone (C4-HSL). Clinical isolate 29, was used for analysis. Control with bovine serum albumin (BSA; 100 μg/mL) is shown as black bar; Ssopox W263I (red bar) and GcL (blue bar) were used at 100 μg/mL. Statistical analysis was performed using Student’s *t* test. GcL effectively hydrolyzes C4-HSL more proficiently than Ssopox W263I. **(B)** AHL detection using *E. coli* sensor plasmid, pSB1075, which detects 3-oxo-dodecanoyl homoserine lactone (3OC12-HSL). Clinical isolate 29, was used for analysis. Control with bovine serum albumin (BSA; 100 μg/mL) is shown as black bar; Ssopox W263I (red bar) and GcL (blue bar) were used at 100 μg/mL. Statistical analysis was performed using Student’s *t* test. GcL and Ssopox W263I hydrolyze 3OC12-HSL with similar rates. **(C)** AHL detection using *E. coli* based sensor plasmid, pSB536, which specifically detects C4-homoserine lactone (C4-HSL). WT strain, PA14, was used for analysis. Control with bovine serum albumin (BSA; 100 μg/mL) is shown as black bar; Ssopox W263I (red bar) and GcL (blue bar) were used at 100 μg/mL. Statistical analysis was performed using Student’s *t* test. GcL effectively hydrolyzes C4-HSL more proficiently than Ssopox W263I. **(D)** AHL detection using *E. coli* sensor plasmid, pSB1075, which detects 3-oxo-dodecanoyl homoserine lactone (3OC12-HSL). WT strain, PA14, was used for analysis. Control with bovine serum albumin (BSA; 100 μg/mL) is shown as black bar; Ssopox W263I (red bar) and GcL (blue bar) were used at 100 μg/mL. Statistical analysis was performed using Student’s *t* test. GcL and Ssopox W263I hydrolyze 3OC12-HSL effectively but GcL is again more proficient. Tests were considered significant for *p* values ≤ 0.05. ^∗^*p* ≤ 0.05, ^∗∗^*p* ≤ 0.01, and ^∗∗∗^*p* ≤ 0.001.

In this study, we used two well-characterized lactonases with distinct substrate specificity. One of the enzymes, GcL, hydrolyzes both C4- and 3-oxo-C12 HSL (Bergonzi et al., 2019), whereas the other lactonase, SsoPox W263I, prefers 3-oxo-C12 HSL (Hiblot et al., 2013; Rémy et al., 2019; see Supplementary Table S2). Therefore, GcL is expected to quench both AHL-based QS circuits in *P. aeruginosa*, and SsoPox W263I will mainly quench the 3-oxo-C12 HSL based QS circuit. In order to measure the hydrolysis of AHLs in culture media by the addition of the two enzymes, we used previously reported biosensors. Specifically, we used the reporter plasmid pSB536 that senses C4-HSL (Swift et al., 1997; Winson et al., 1998), transformed in *E. coli* cells, to quantify the C4-HSL in culture supernatants of *P. aeruginosa* clinical isolate “29” and PA14 (Figures 1A,C). Consistent with the reported substrate preference of both lactonases, treatment with GcL dramatically reduced the concentration of C4-HSL in culture supernatants, whereas the treatment with SsoPox did only slightly reduce it. In order to quantify the second AHL produced by *P. aeruginosa*, we used another reporter plasmid, pSB1075, specific to 3-oxo-C12 HSL (Swift et al., 1997; Winson et al., 1998), which was also transformed into *E. coli*. Amounts of 3-oxo-C12 HSL were significantly reduced for both lactonase treatments (GcL and SsoPox), as compared to the control (Figures 1B,D). We note three points: (i) the concentration of AHLs produced by the two *P. aeruginosas* vary, but is high (in the hundreds of nM range for both C4- and 3-oxo-C12 HSL), and measurements are similar to previous studies with *P. aeruginosa* cultures (Smith, 2003), (ii) the observed AHL degradation is consistent with the reported substrate preference of both enzymes, and (iii) in the tested conditions, the used lactonases nearly completely degrade AHLs produced by PA14, but while it does decrease AHLs concentration significantly in the case of isolate 29, it does not lead to the complete removal of AHLs in supernatants. This may be due to more organized, and less distorted regulation of the QS hierarchy in the wild-type strain as compared to a lung isolate, as previously described by Feltner et al. (2016).

In addition, we performed a time-course evaluation using these biosensors to detect short and long-chain AHLs and the ability of the two enzymes to hydrolyze each substrate (Supplementary Figure S8). Similarly, this analysis reveals that Ssopox W263I exhibits very little activity against C4-HSL, contrary to GcL, and that both enzymes degrade 3-oxo C12 HSL similarly (Supplementary Figure S7). This, together with controlling the stability of the lactonases over time in the cell culture conditions (Supplementary Figure S9) demonstrates the differential ability of AHL degradation of both enzymes in the tested conditions.

### Treatments With Both Lactonases Are Not Cytotoxic and Led to Inhibition of Biofilm in *Pseudomonas aeruginosa*

**FIGURE 2:**
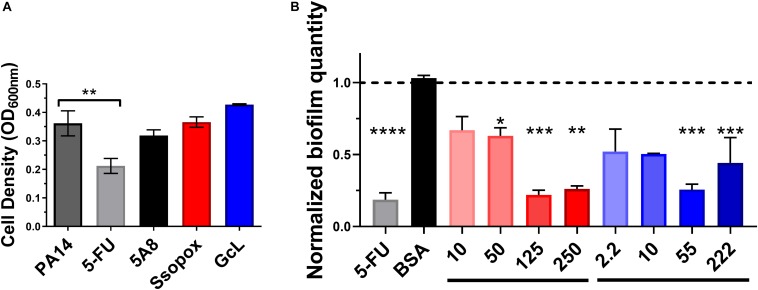
Lactonase treatment is not cytotoxic, acts in a dose dependent manner, and is effective at inhibiting biofilm formation in *Pseudomonas aeruginosa*. **(A)** Cell density of *P. aeruginosa* PA14, without treatment (control; dark gray bar), or with addition of 5-FU (5′-fluorouracil; 60 μM; light gray bar), SsoPox mutant 5A8 (an inactive lactonase; 125 μg/mL; black bar), Ssopox W263I (a lactonase; 125 μg/mL; red bar), or GcL (a lactonase; 55 μg/mL; blue bar). Statistical analysis was performed using Student’s *t* test. **(B)** Normalized PA14 biofilm quantity as quantified by Crystal Violet assay, using varying concentrations of Ssopox W263I (red bars), GcL (blue bars), or controls 5-FU (fluorouracil; 60 μM; gray bar), or BSA (bovine serum albumin; 100 μg/mL; black bar). Concentrations of Ssopox W263I and GcL are μg/mL. Statistical analysis was performed using Student’s *t* test. ^∗^ indicate statistical significance. Maximal inhibition of biofilm was at 125 μg/mL for Ssopox, and 55 μg/mL for GcL.

We evaluated the effects of lactonase treatments on the growth and biofilm formation of *P. aeruginosa* strain PA14 (Figure 2). PA14 is a virulent strain that causes disease in a wide range of organisms, including humans (Mikkelsen et al., 2011). Experiments reveal that treatments with both lactonases, and an inactive variant of SsoPox (5A8), previously described (Bergonzi et al., 2018), do not alter the cell density (Figure 2A). However, treatment with 5-FU, a compound described as a QS inhibitor and which made it to clinical trials for its anti-biofilm forming properties (Walz et al., 2010), was found to be cytotoxic. Cytotoxicity is especially relevant as the ability to kill cells by any potential quorum quenching therapy may increase pressure to acquire resistance, such as that seen with 5-FU and the brominated furanone, C-30. Therefore, the use of quorum quenching enzymes may be advantageous to the use of QSIs as they perhaps may be less prone to lead to bacterial resistance (García-Contreras et al., 2013, 2015; Guendouze et al., 2017).

The ability of SsoPox W263I to inhibit *P. aeruginosa* biofilm formation was previously reported, including on diabetic foot ulcer isolates (Guendouze et al., 2017). Here, we obtain similar inhibitory patterns for PA14, with maximal observed biofilm inhibition (78%) with 125 μg/mL of Ssopox W263I. This observed inhibition level is similar to that observed with 5-FU, yet some of the biofilm inhibitory activity of the latter compound may be the outcome of its observed cytotoxicity. We show that the lactonase, GcL, is also capable of inhibiting biofilm formation, with a maximal potency observed with 55 μg/mL of enzyme (74% inhibition) (Figure 2B). In fact, the optimal concentrations for both lactonases led to inhibitory levels that are not significantly different from each other, and these respective concentrations were used for the rest of the study. Notably, while the inhibition of biofilm formation increased with the dose of enzyme, it does not lead to full inhibition, and biofilm amount tends to increase at higher lactonase concentration. Similar observations were made with other pathogens (Bergonzi et al., 2019), and this may suggest that the response to AHL concentration in the culture media is not linear.

Testing the effects of the two lactonases on PA14 knock-out strains, including Δ*lasR*,Δ*rhlR, and*Δ*rhlI* (Supplementary Figure S10), we further confirmed that the phenotype changes associated with the lactonases are related to QS circuits. Indeed, while both biofilm and pyocyanin are inhibited by both lactonases in PA14, there are no significant changes in phenotypes for the three mutant strains with or without lactonase treatments.

### Acyl Homoserine Lactone Signal Disruption Using Lactonases Inhibits Biofilm and Virulence Factor Production in CF-Adapted *Pseudomonas aeruginosa* Isolates

39 CF-adapted *P. aeruginosa* clinical isolates were used in this study and characterized for their drug resistance to either cefepime, levofloxacin, piperacillin-tazobactam, commonly used antibiotics for treating CF-patients (Supplementary Figure S1). Only one isolate was found to be multi-resistant (61), two others were showing an intermediate-level of resistance for multiple antibiotics (58 and 63), and 8 other isolates showed levels of resistance to one tested antibiotic (19, 27, 29, 33, 50, 56, 64, and 66). Overall, 11 out of 39 isolates (28%) showed at least intermediate level of resistance for one or more of the tested antibiotics. We tested for the presence of the *lasr* gene in these isolates using PCR amplification (Supplementary Table S1). This method does not inform on the activity of *the lasr* gene, nor about any potential mutations carried by *the lasr* gene. Amplification results can, however, suggest that these isolates may show variability regarding *the lasr* gene. Indeed, *the lasr* amplification was negative for 11 isolates (out of 39; 28%) and the latter may therefore be *lasr*-null (27, 32, 54, 55, 58, 61, 62, 64, 70, 74, and 75). CF-adapted isolates were previously reported to frequently harbor mutations or deletion of the *lasr* gene, and these changes are associated with hyperinflammation and disease progression (Hoffman et al., 2009; LaFayette et al., 2015), and previously observed in CF-adapted *P. aeruginosa* isolates (D’Argenio et al., 2007; Dekimpe and Déziel, 2009; Bjarnsholt et al., 2010).

**FIGURE 3:**
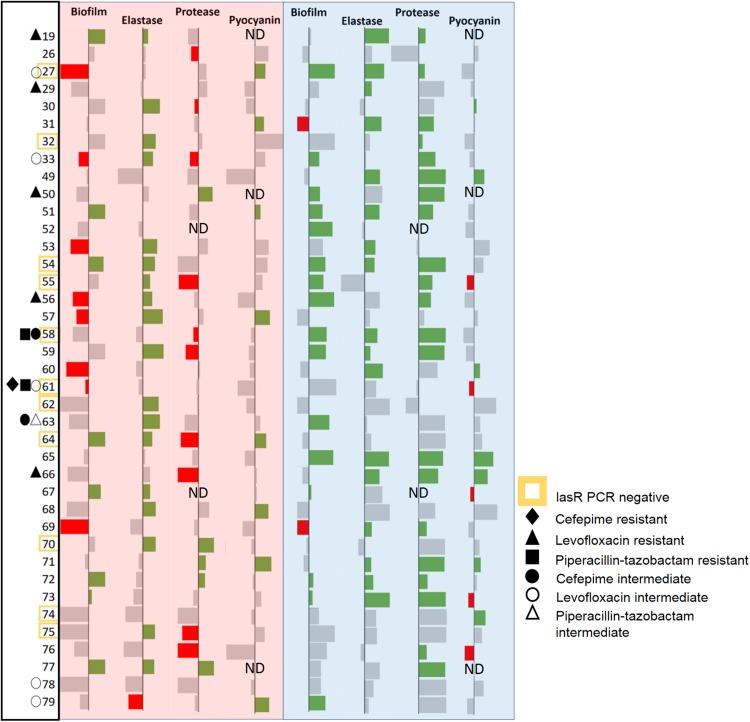
Data bar table showing effectiveness of lactonase treatment on measured virulence factors (VFs). Data bar table showing the effects of Ssopox (red background) or GcL (blue background) on four virulence factors produced by *P. aeruginosa* clinical isolates (39), including biofilm, elastase, protease, pyocyanin. Data bars show inhibition (rightward deflection) or stimulation (leftward deflection). Statistically significant deflections (*p* < 0.05 using Student’s *t* test) are shown in green (inhibition) or red (stimulation). Strain numbers are listed on the left. Negative PCR result for the *lasr* gene is designated by a yellow square around the strain name. The resistance pattern of each clinical isolate is also designated. ND indicates “not determined.”

These isolates were treated with the lactonases, GcL or SsoPox W263I, and the effects of signal disruption were evaluated by monitoring the production of key virulence factors, including elastase, protease, and pyocyanin, and biofilm formation (Figure 3 and Supplementary Figures S2, S3). We note that given the effects of both lactonases on AHLs concentrations in cultures (Figure 1 and Supplementary Figure S8), measured phenotype changes are concomitant with differential degradation of C4 and 3-oxo C12 HSL. Results show that the majority of the tested isolates (36/39; 92%) are inhibited for one of the measured factors by at least one of the enzymes. This result is intriguing, since CF-adapted *P. aeruginosa* isolates are known for their propensity to remodel their QS systems (D’Argenio et al., 2007; Bjarnsholt et al., 2010). Yet, it is consistent with reports highlighting the importance of QS in lung infections, including chronic and late-stage infections (Winstanley and Fothergill, 2009; Bjarnsholt et al., 2010). Notably, in the case of several isolates, the enzymatic treatment significantly increased the production of some measured factors. These cases are infrequent for the GcL treated isolates (7/39; 18%), but were observed more for those treated with SsoPox treatment (19/39; 49%). This phenomenon was more commonly seen for biofilm and protease production, both being the products of more complex regulation systems (Parsek and Greenberg, 2004; Mellbye and Schuster, 2014) which involve but are not entirely reliant upon QS, unlike that of elastase and pyocyanin. In these cases, the stimulation of a factor is typically concomitant to the inhibition of other measured factors (11/19 for SsoPox, 6/7 for GcL; 17/26; 65% overall). Alternatively, several observations between virulence products are noted: inhibition of elastase and protease (Spearman coefficient, *r* = 0.2359, and *p* = 0.0119), and protease and pyocyanin (Spearman coefficient, *r* = 0.2251, *p* = 0.0216) are significantly correlated with the treatment of Ssopox (Supplementary Figure S4). With GcL treatment, only biofilm and pyocyanin inhibition are significantly correlated (Spearman coefficient, *r* = −0.1893, *p* = 0.0497; Supplementary Figure S5). These large differences between the lactonase treatment outcomes may relate to different QS regulatory circuits within these clinical isolates. For example, CF-adapted *P. aeruginosa* isolates can lose or mutate the LasR system (D’Argenio et al., 2007) and/or harbor a RhlR system that is independent of the LasR system (Feltner et al., 2016; Kostylev et al., 2019). Such remodeling of QS systems could account for the observed superior consistency in the inhibitory effect by the lactonase, GcL.

We note also that out of 11 isolates for which the *lasr* amplification was negative (possibly *lasr*-null), 3 isolates (61, 74, and 58) are not inhibited by the lactonase degrading 3-oxo-C12 HSL (SsoPox W263I), and 5 (61, 62, 64, 70, and 75) are not inhibited by the other lactonase (GcL). The explanation for the ability of SsoPox to inhibit *lasr*-null strains is unclear, but may originate from the weak activity of the SsoPox enzyme against C4-HSL and/or the high degree of remodeling of QS circuits in the studied isolates.

### Lactonases With Distinct Acyl Homoserine Lactone Preferences Have Differential Effects on CF-Adapted *Pseudomonas aeruginosa* Isolates

**FIGURE 4:**
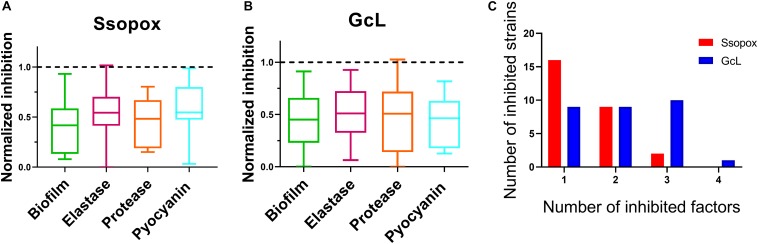
Both lactonases effectively inhibit virulence factors (VFs), but GcL simultaneously inhibits more VFs than SsoPox W263I in *P. aeruginosa* isolates. Box plots showing the reduction of all statistically significant (*p* < 0.05) virulence factors on clinical isolates of *P. aeruginosa* in response to Ssopox W263I **(A)** or GcL **(B)**, and as compared to control treatment. Ssopox W263I concentration was 125 μg/mL; GcL concentration was 55 μg/mL. **(C)** Distribution of inhibited strains for one or more virulence factors for the two lactonase treatments.

Analysis of inhibitory levels for both treatments reveal that both enzymes exhibit a similar inhibitory potency against the clinical isolates. The box plots show an average biofilm inhibition of 55% for both enzymes across all tested isolates; 48% for elastase inhibition; 53% and 49% for protease and pyocyanin inhibition, respectively (Figures 4A,B). As shown in the bar graphs, when considering only the isolates that are sensitive to the lactonases, the degree of inhibition of each virulence factor by each lactonase is similar; inhibition levels of biofilm are 57% and 54%, for elastase, 47% and 49%, for protease 55% and 52%, and for pyocyanin, 44% and 54%, all for Ssopox and GcL, respectively (Supplementary Figure S6). However, both lactonase treatments result in a different average number of inhibited factors per isolates: 1.5 ± 0.6 for SsoPox, and 2 ± 1 for GcL (Figure 4C).

**FIGURE 5:**
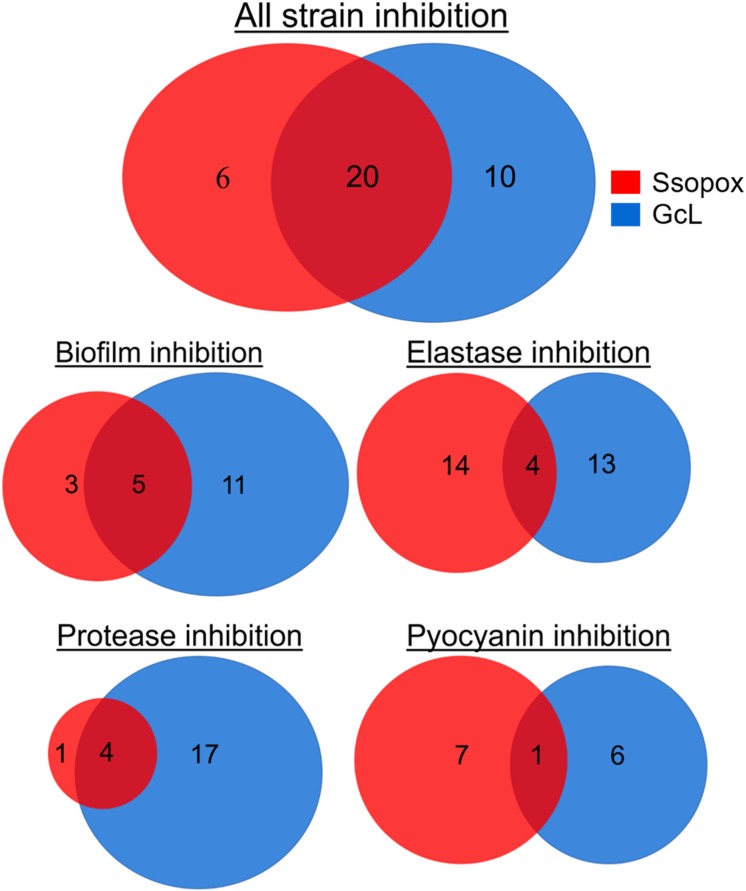
Venn diagrams representing the observed inhibition of VFs by the two lactonase treatments. Venn diagrams showing the effects of treatment with Ssopox (red) or GcL (blue) on individual virulence factors. Ssopox concentration was 125 μg/mL; GcL concentration was 55 μg/mL. This diagram shows that 30/39 and 26/39 strains were inhibited for at least one VF with GcL and SsoPox W263I treatments, respectively. Interestingly, while there is a significant overlap between the inhibitory activities of both lactonases (20 strains, over a total of 36 strains inhibited, 55%), there are in fact a significant number of strains that are inhibited by only one lactonase (16 strains, 44% of 36).

In fact, treatment with the lactonase SsoPox W263I led to the inhibition of 26 isolates (out of 39; 67%), whereas treatment with GcL affected 30 isolates (out of 39; 77%). While there is overlap between the inhibitory activities of both lactonases (20 strains, over a total of 36 strains inhibited, 55%), there are in fact a significant number of strains that are inhibited by only one lactonase (16 strains, 44% of 36) (Figure 5). Treatments with each enzyme resulted in a similar number of isolates to be inhibited for elastase production (18 for SsoPox, 17 for GcL) and pyocyanin inhibition (8 for SsoPox, 7 for GcL). However, GcL treatments resulted in more isolates to be inhibited for biofilm formation (16 for GcL, 8 for SsoPox) and for secreted proteases (21 for GcL, 5 for SsoPox) (Figure 5). Altogether, these differences in treatments suggest that the AHL preference of the lactonase determines the activity spectrum of the enzyme and the number of inhibited virulence factors, but not the inhibition levels of the latter.

## Conclusion

Focusing on 39 clinical isolates of *P. aeruginosa* from CF-patients, including drug resistant strains, we investigated the ability of two quorum quenching lactonases with distinct AHL preference to inhibit virulence factors and biofilm formation. Interestingly, we show that the majority of tested isolates are inhibited by lactonase treatment (92%), despite the ability of CF-adapted *P. aeruginosa* isolates to remodel their QS circuits (D’Argenio et al., 2007; Bjarnsholt et al., 2010). We find that some isolates are inhibited by both enzymes (56%), whereas a significant number of strains were inhibited by only one lactonase (44%). In fact, the broad spectrum lactonase (GcL) inhibited 77% of the isolates, whereas the specific SsoPox, targeting primarily 3-oxo-C12 HSL, inhibited 67% of them. The distinct inhibitory profile of the two enzymes is also evidenced by the average number of inhibited virulence factors per isolate (2 and 1.5, for GcL and Ssopox, respectively). The overall superior inhibitory activity of the broad spectrum lactonase, GcL, may originate from its ability to degrade C4-HSL and 3-oxo-C12 HSL and the ability of CF-adapted *P. aeruginosa* isolates to harbor a LasR-independent RhlR system (Kostylev et al., 2019). These data show the inability to fully quench QS signaling in bacteria by targeting a single communication molecule. Since the interplay of multiple systems of signal production and feedback affect virulence and other factor production, particularly in clinical isolates, multiple quorum quenching enzymes with different substrate targets may be required to proficiently disrupt bacterial communication.

## Data Availability Statement

All datasets generated for this study are included in the article/Supplementary Material.

## Author Contributions

ME conceived and designed the work. KM, RM, IL, and RS performed the experiments. ME, KM, RM, IL, and RS analyzed the data. ME and KM wrote the first draft. ME, KM, RS, and JD wrote sections of the manuscript. ME, KM, and JD critically revised the manuscript. All authors read and approved the final manuscript.

## Conflict of Interest

ME has a patent WO2014167140 A1 licensed to Gene&GreenTK. The remaining authors declare that the research was conducted in the absence of any commercial or financial relationships that could be construed as a potential conflict of interest.
