# Supplementary material for: Effects of Signal Disruption Depends on the Substrate Preference of the Lactonase
Source: Front Microbiol. 2020 Jan 14;10:3003. doi: 10.3389/fmicb.2019.03003 (PMC6971184; doi:10.3389/fmicb.2019.03003)
Supplement: Supplementary file 1 [file Data_Sheet_1.PDF]

Supplementary Material

Effects of Signal Disruption Depends on the Substrate Preference of the Lactonase.

Kathleen Mahan[a], Ryan Martinmaki[b], Isabel Larus[b], Rakesh Sikdar[b], Jordan Dunitz[a,c] and Mikael Elias[b]*

[a] Department of Medicine, Division of Pulmonary, Allergy, Critical Care and Sleep Medicine, University of Minnesota, Minneapolis, Minnesota, 55455, USA

[b] Biochemistry, Molecular Biology & Biophysics Department and BioTechnology Institute, University of Minnesota, Saint Paul, Minnesota, 55108, USA

[c] Department of Medicine and Minnesota Cystic Fibrosis Center and Adult CF Program, University of Minnesota, Minneapolis, Minnesota, 55455, USA

Correspondence email: Prof. Mikael Elias; Tel: +1-612 626 1915; Fax: +1 612 625 5780; mhelias@umn.edu

Table of Contents for Supplementary Materials

Supplementary Page 2 – Table S1 - *lasr* PCR results of clinical isolates

Supplementary Page 3 – Figure S1 – Antibiotic resistance patterns of the clinical isolates

Supplementary Page 4 – Figure S2 – Clinical isolates of *Pseudomonas aeruginosa* inhibited by SsoPox

Supplementary Page 5 – Figure S3 - Clinical isolates of *Pseudomonas aeruginosa* inhibited by GcL

Supplementary Page 6 – Figure S4 – Correlation plot data of SsoPox treatments

Supplementary Page 7 – Figure S5 – Correlation plot data of GcL treatments

Supplementary Page 8 – Figure S6 – Summary graph of efficacy of SsoPox and GcL on virulence factors

Supplementary Page 9 –Figure S7 – Enzyme activity graph of Ssopox and. GcL on C4-HSL vs. 3OC12-HSL

Supplementary Page 10 – Figure S8 – Biosensor time-course data for short vs. long-chain AHLs

Supplementary Page 11 – Figure S9 – Enzyme (Ssopox and GcL) activity over time

Supplementary Page 12 – Figure S10 - Virulence factors and inhibition using lactonases on mutant strains

Supplementary Page 13 – Table S2 – Catalytic efficiency of each lactonase on AHLs

Supplementary Page 14 – Table S3 – Summary of strains and primers used

Supplementary Page 15 – References for Table S3

Supplementary Page 13 - Methods – Enzyme activity assay method

Pseudomonas aeruginosa strain	lasR PCR	*Pseudomonas aeruginosa* strain	lasR PCR
**19**	Positive	61	Negative
**26**	Positive	62	Negative
**27**	Negative	63	Positive
**29**	Positive	64	Negative
**30**	Positive	65	Positive
**31**	Positive	66	Positive
**32**	Negative	67	Positive
**33**	Positive	68	Positive
**49**	Positive	69	Positive
**50**	Positive	70	Negative
**51**	Positive	71	Positive
**52**	Positive	72	Positive
**53**	Positive	73	Positive
**54**	Negative	74	Negative
**55**	Negative	75	Negative
**56**	Positive	76	Positive
**57**	Positive	77	Positive
**58**	Negative	78	Positive
**59**	Positive	79	Positive
**60**	Positive		

Table S1. PCR results of the presence of the *lasr* gene in clinical isolates. Table displaying PCR results for the presence of *lasr* gene in each clinical isolate. 28/39 strains had a positive PCR result after visualization of PCR products on an agar gel.

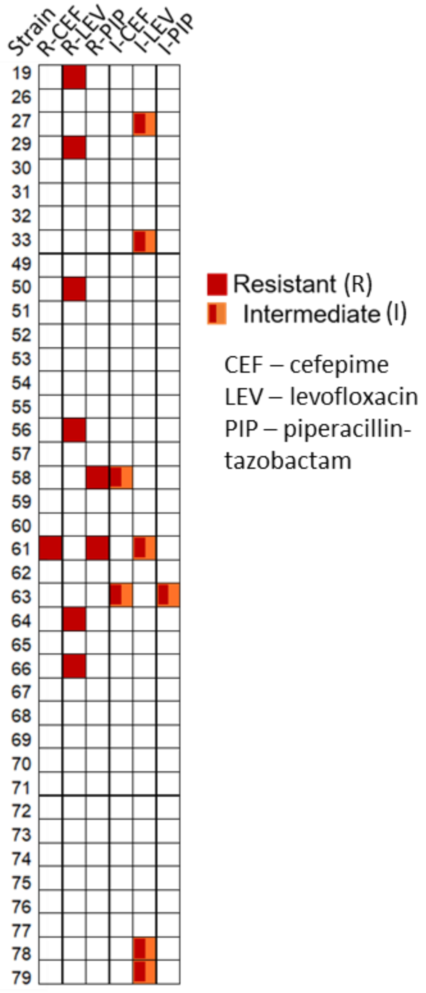

Figure S1. Resistance pattern of each clinical isolate to commonly used antibiotics. Resistance patterns of each strain are shown in response to cefepime (CEF), levofloxacin (LEV), or piperacillin-tazobactam (PIP) based on Clinical & Laboratory Standards Institute (CLSI) zone of inhibition (ZOI) guidelines. (R) – resistant; (I) – intermediate. Each strain is sensitive to each tested antibiotic unless otherwise designated.

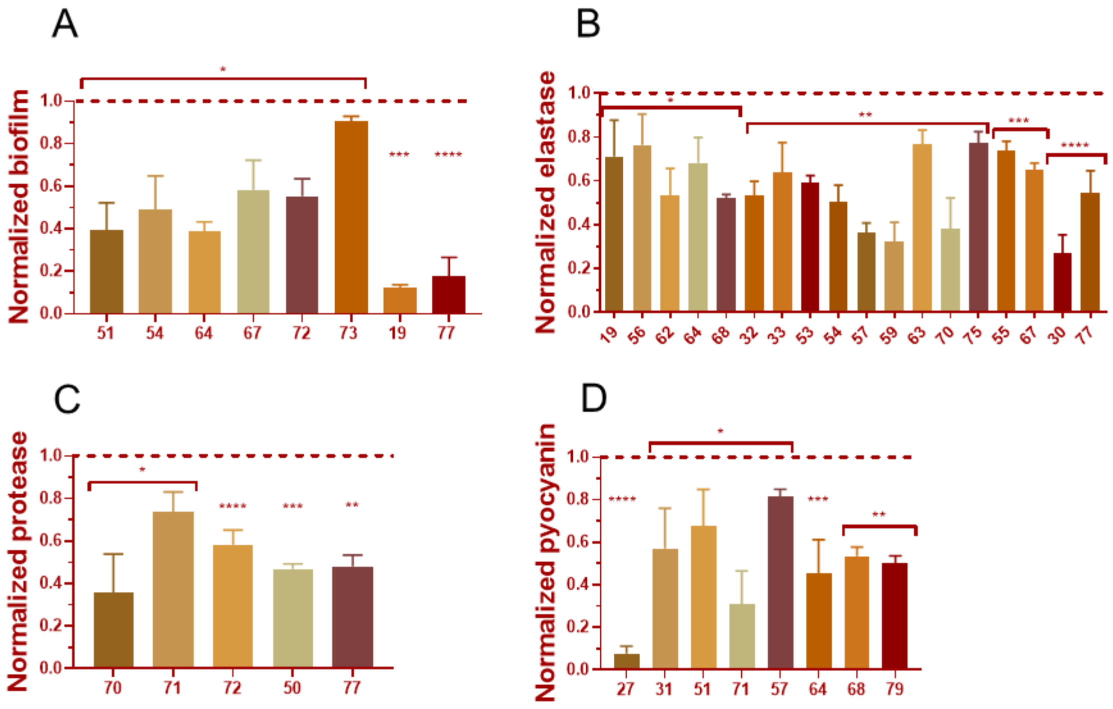

Figure S2. Ssopox reduces virulence factor or biofilm production in clinical isolates. Clinical isolates with statistically significant reduction in biofilm quantity (**A**), elastase activity (**B**), protease activity (**C**), or pyocyanin production (**D**) with treatment of Ssopox. All values are normalized to values of untreated clinical strain (control). Ssopox concentration used was 125 µg/ml. Statistical analysis was performed using Student's t test. *indicate degree of statistical significance.

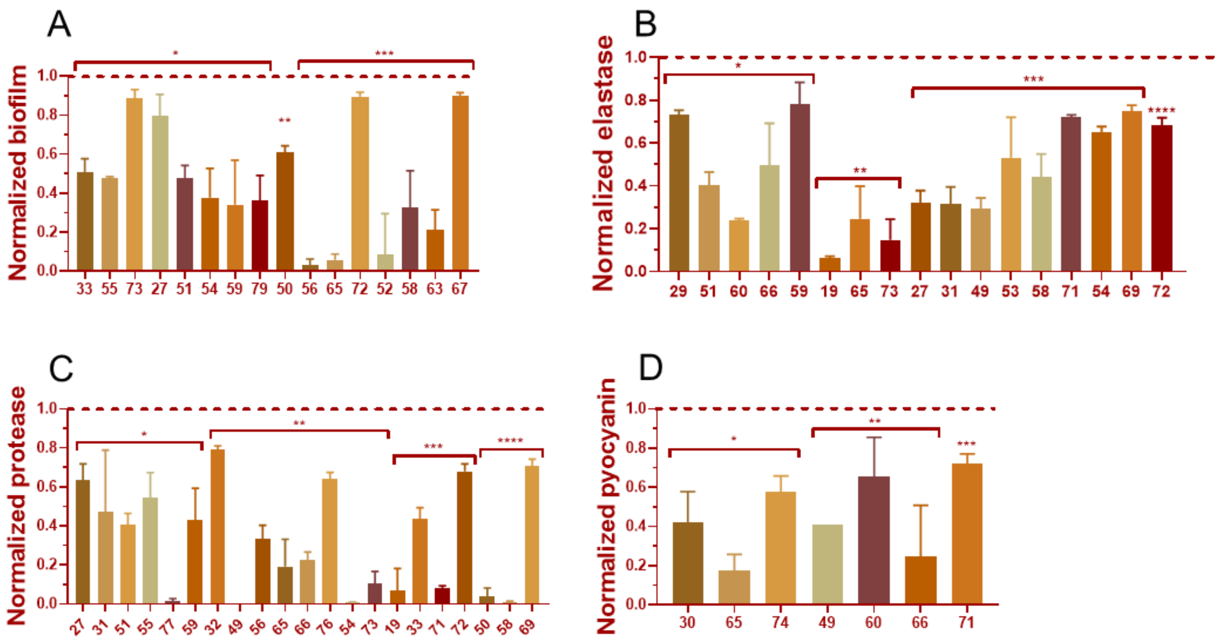

Figure S3. GcL reduces virulence factor or biofilm production in clinical isolates. A, B, C. D. Clinical isolates with statistically significant reduction in biofilm quantity (**A**), elastase activity (**B**), protease activity (**C**), or pyocyanin production (**D**) with treatment of GcL. All values are normalized to values of untreated clinical strain, control (labeled). GcL concentration used was 55 µg/ml. Statistical analysis was performed using Student's t test. *indicate degree of statistical significance.

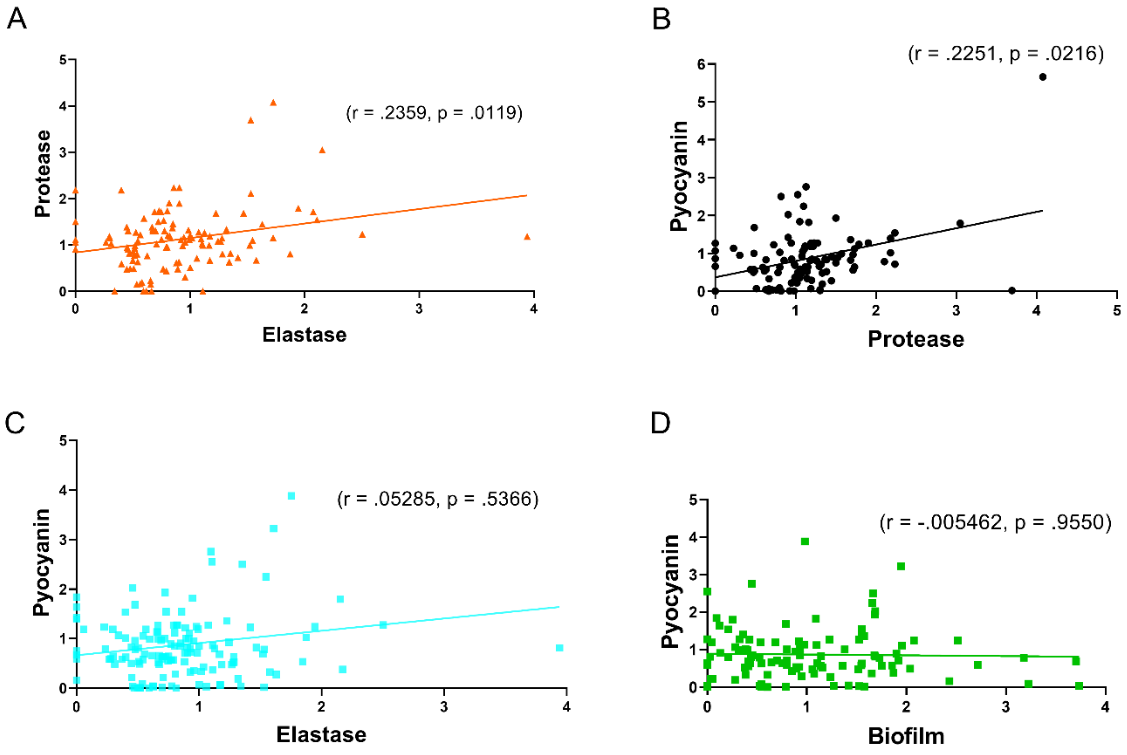

Figure S4. Associated virulence factor inhibition by Ssopox in clinical isolates of *Pseudomonas aeruginosa.* Correlation plots showing the association between virulence factors produced by clinical isolates after treatment with Ssopox treatment (125 µg/ml). Associations between tested virulence factors may suggest predictive value of a lactonase to disrupt specific quorum sensing circuits causing inhibition of multiple factors. r = Spearman coefficient. Statistically significant associations are indicated with $p \leq 0.05$, figures *A*, and *B*.

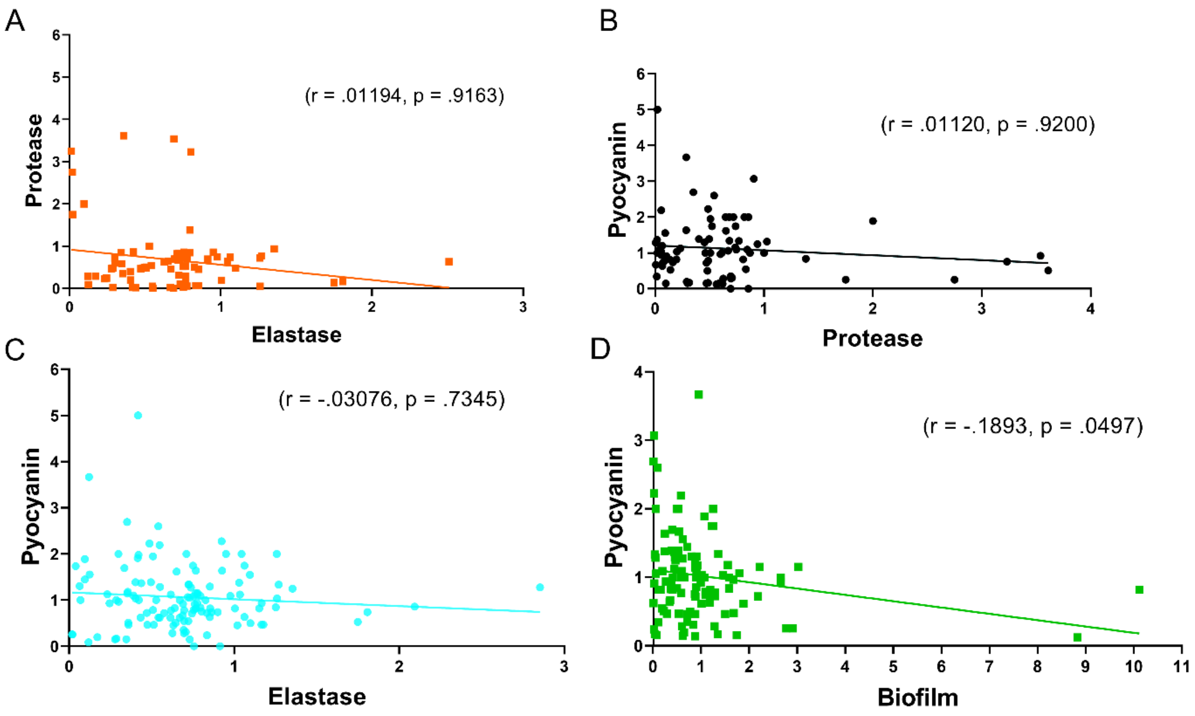

Figure S5. Associated virulence factor inhibition by GcL in clinical isolates of *Pseudomonas aeruginosa*. Correlation plots showing the association between virulence factors produced by clinical isolates after treatment with GcL treatment (55 µg/ml). Associations between tested virulence factors may suggest predictive value of a lactonase to disrupt specific quorum sensing circuits causing inhibition of multiple factors. r = Spearman coefficient. Statistically significant associations are indicated with $p \leq 0.05$, figure *D*.

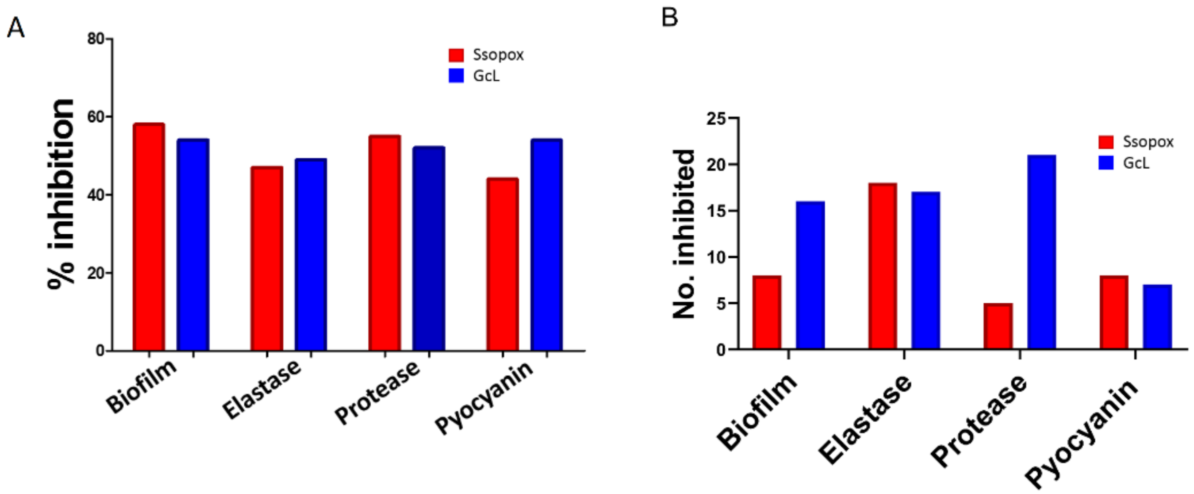

Figure S6. Bar graphs showing inhibitory effects of each lactonase (**A**) The average percentage of inhibition of virulence factors and biofilm is shown for both enzymes, (**B**) The number of isolates inhibited for each measured virulence factor is shown. GcL, the less specific enzyme, is effective at inhibiting more overall VFs as well as causing more overall inhibition for each VF (Ssopox 47% vs. GcL 55%), particularly for protease and biofilm.

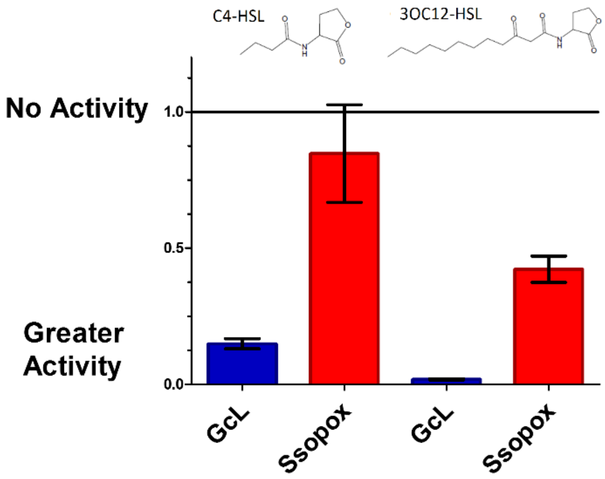

Figure S7. Substrate preference of each lactonase

Acyl homoserine lactone (AHL) detection using E. coli based sensor plasmid, pSB536, which specifically detects C4-homoserine lactone (C4-HSL) or pSB1075, which specifically detects 3OC12-homoserine lactone (3OC12-HSL). *Pseudomonas aeruginosa* WT strain, PA14, was used for analysis. Ssopox W263I (red bar) and GcL (blue bar) were used at 100 µg/ml. GcL effectively hydrolyzes C4-HSL more proficiently than Ssopox W263I whereas both enzymes are effective at hydrolysis of the long-chain AHL, 3OC12-HSL.

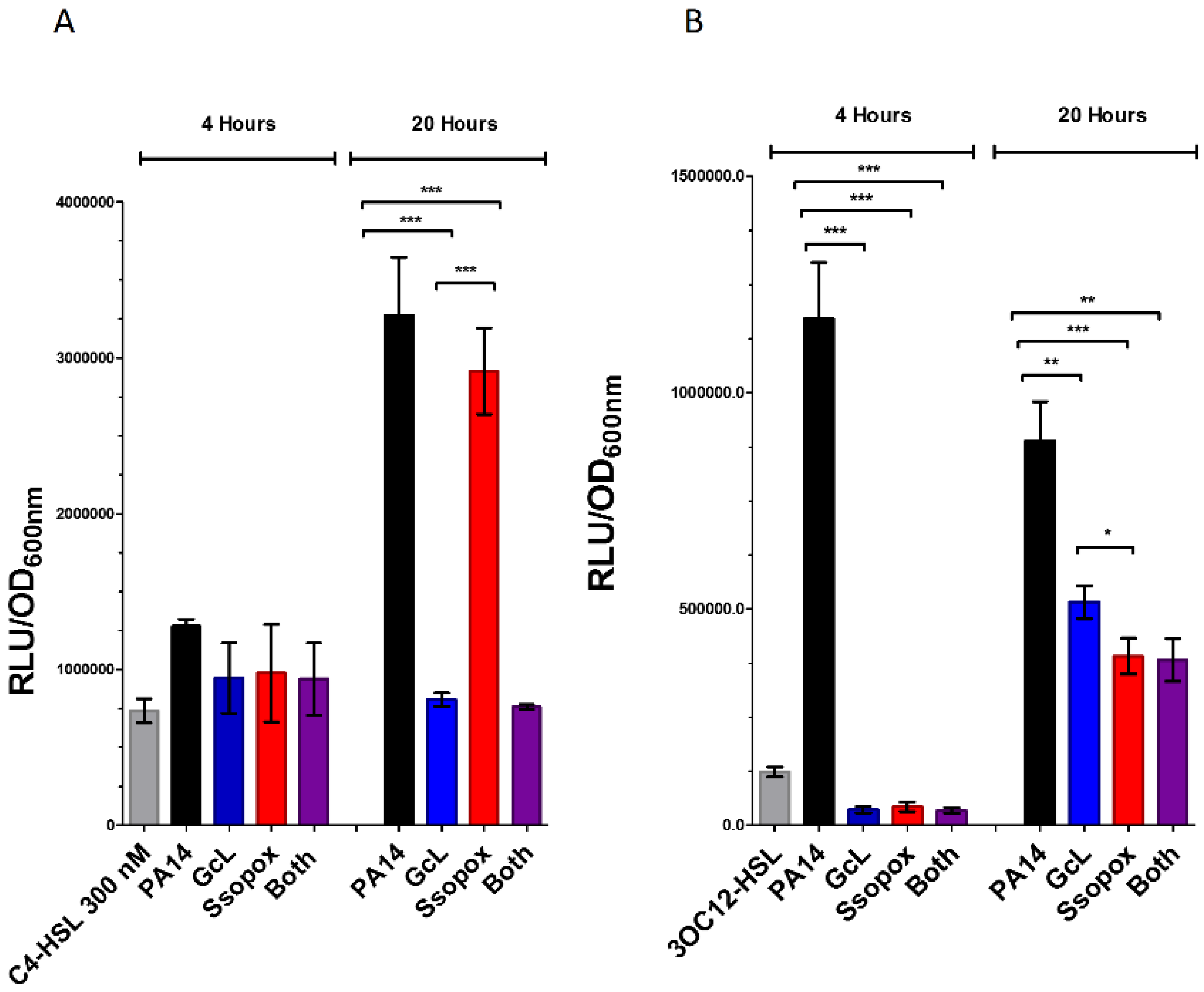

Figure S8. Short and long-chain production of AHLs over time

(A) Acyl homoserine lactone (AHL) detection using *E. coli* based sensor plasmid, pSB536, which specifically detects C4-homoserine lactone (C4-HSL). This assay was performed using supernatant isolated at 4 hours and 20 hours of the assay. *Pseudomonas aeruginosa* WT strain, PA14, was used for analysis. Untreated (PA14) is shown as black bar; Ssopox W263I (red bar), GcL (blue bar) were used at 100 µg/mL; combination of the two lactonases shown in purple. Statistical analysis was performed using Student's t test.

(B) Acyl homoserine lactonase (AHL) detection using *E. coli* sensor plasmid, pSB1075, which detects 3-oxo-dodecanoyl homoserine lactone (3OC12-HSL). This assay was performed using supernatant isolated at 4 hours and 20 hours of the assay. *Pseudomonas aeruginosa* WT strain, PA14, was used for analysis. Untreated (PA14) is shown as black bar; Ssopox W263I (red bar), GcL (blue bar) were used at 100 µg/mL; combination of the two lactonases shown in purple. Statistical analysis was performed using Student's t test.

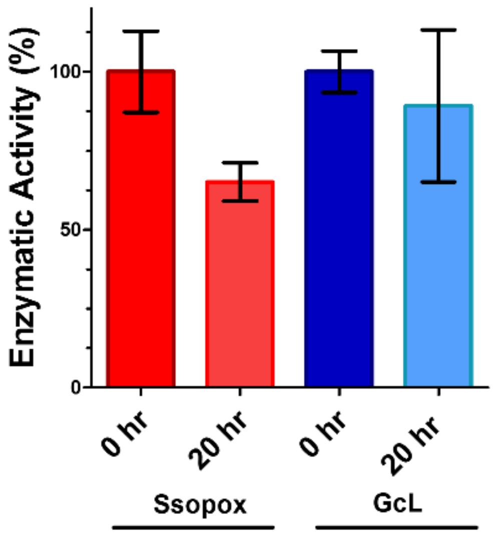

Figure S9. Lactonases Ssopox W263I and GcL remain active after 20 hours of incubation at 37˚ C in LB media.

Ssopox W263I (red) and GcL (blue) activity were determined at 0 hours and at 20 hours after incubation with Luria-Bertani (LB) medium at 37˚ C (expressed in percentage from baseline). There is minimal reduction in activity of the two enzymes over this incubation period corresponding to significant thermostability at an elevated temperature.

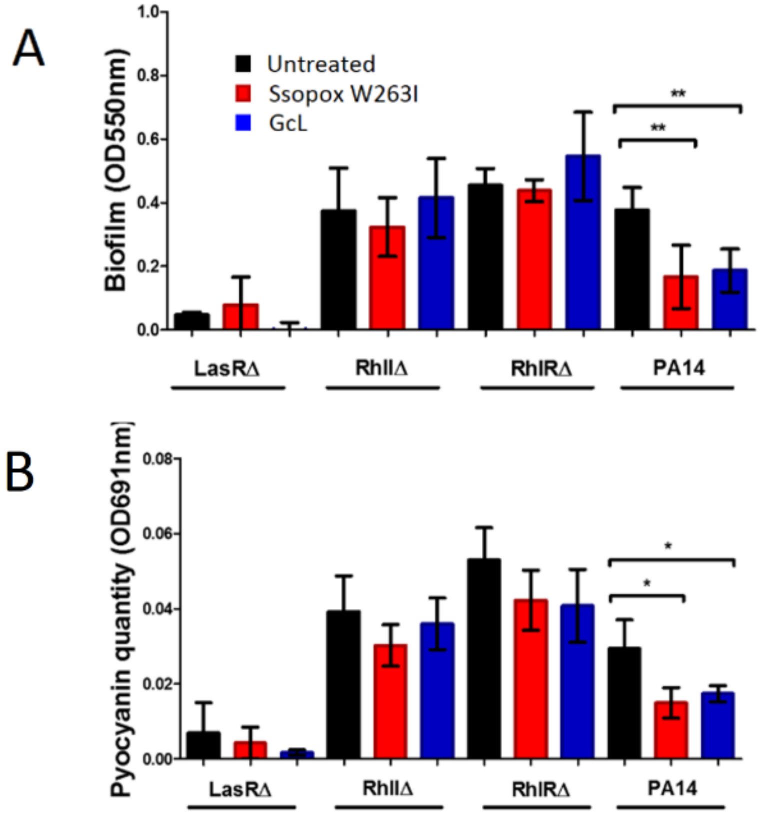

Figure S10. Virulence factors and inhibition using lactonases on mutant strains

(A), (B), (C) Level of inhibition of virulence factors, including biofilm, and pyocyanin for the *Pseudomonas aeruginosa* WT strain, PA14, LasRΔ, RhlIΔ, RhlRΔ. Control is untreated, (shown in black), while concentration for Ssopox W263I (shown in red) was 125 µg/ml, and GcL (shown in blue) concentration was 55 µg/ml. Statistical analysis was performed using Student's t test. * indicate statistical significance.

Table S2. Catalytic efficiency of each lactonase.

Enzyme	*Sso*Pox W263I		*Gc*L	
AHL	**3-oxo-C_{12} HSL[a]**	**C_4 HSL[b]**	**3-oxo-C_{12} HSL[c]**	**C_4 HSL[b]**
K_M (µM)	17.8±4.9	(4.3±0.1)x10^3	97±9	229±57
k_{cat} (s^{-1})	1.8±0.1	0.37±0.01	10±1	19.1±1.5
k_{cat}/K_M ($M^{-1}.s^{-1}$)	(1±0.3)x10^5	86±1	(1.1±0.1)x10^5	(8.3±2.2)x10^4
Preference for 3-oxo-C12 HSL[§]	1,163		1.3	
Catalytic efficiency ratio[¶]	N/A	N/A	1.1	965

§ Ratio of catalytic efficiency for 3-oxo C12 HSL and catalytic efficiency for C4 HSL

¶ Ratio of catalytic efficiencies of GcL over catalytic efficiencies of SsoPox W263I.

.

Table S3. Bacterial strains and primers used in the study.

Bacterial strain	Description	Reference or source
<u>*P. aeruginosa* strains</u>		
PA14	Wild-type strain, generous gift from Dr. Ryan Hunter University of Minnesota, Minneapolis, MN	Laboratory stock
PA14 Δ*lasR*	PA14 containing an in-frame *lasR* deletion, generous gift from Ausubel Lab, Harvard Medical School, Boston, MA	(1)
SM32 Δ*rhlR*	PA14 containing an in-frame *rhlR* deletion, generous gift from Bassler Lab, Princeton University, Princeton, NJ	(2)
SM52 Δ*rhlI*	PA14 containing an in-frame *rhlI* deletion, generous gift from Bassler Lab, Princeton University, Princeton, NJ	(2)
39 *Pseudomonas aeruginosa* clinical isolates	Generous gift from Dr. Ryan Hunter University of Minnesota, Minneapolis, MN	Laboratory stock
<u>*E. coli* strains</u>		
pSB536	JM109 cells containing pUC18 plasmid with a fusion of rhlRI’::luxCDABE as a bioluminescent reporter, generous gift from Dr. Olga Zaborina, University of Chicago	(3) (4)
pSB1075	S17 cells containing pUC18 plasmid with a fusion of lasRI′::luxCDABE as a bioluminescent reporter, generous gift from Dr. Olga Zaborina, University of Chicago	(3) (4)
OP50	Common *C. elegans* food source kindly provided by the CGC, University of Minnesota, Minneapolis, MN	(5)
Primers		
las1 (upstream of *lasr)*	5’ – CGCCGAACTGGAAAAGTGGC - 3’	(6)

las2 (downstream of *lasr)* 5’ – TGAGAGGCAAGATCAGAGAG – 3’ (6)

References:

1. Feinbaum, R., Urbach, J., Liberati, N., Djonovic, S., Adonizio, A., Carvunis, A., and Ausubel, F. (2012). Genome-wide identification of *Pseudomonas aeruginosa* virulence-related genes using a *Caenorhabditis elegans* infection model. *PLOS.* 8, 1-22. doi: 10.1371/journal.ppat.1002813.

2. Hoyland-Kroghsbo, N., Paczkowski, J., Mukherjee, S., Broniewski, J., Westra, E., Bondy-Denomy, J., Bassler, B. (2017). Quorum sensing controls the *Pseudomonas aeruginosa* CRISPR-Cas adaptive immune system. *Proc. Natl. Acad. Sci. USA.* 114(1): 131–135. doi: 10.1073/pnas.1617415113.

3. Swift, S., Karlyshev, A. V., Fish, L., Durant, E. L., Winson, M. K., Chhabra, S. R., et al. (1997). Quorum sensing in Aeromonas hydrophila and Aeromonas salmonicida: identification of the LuxRI homologs AhyRI and AsaRI and their cognate N-acylhomoserine lactone signal molecules*. J. Bacteriol*. 179, 5271–5281. doi:10.1128/jb.179.17.5271-5281.1997.

4. Winson, M. K., Swift, S., Fish, L., Throup, J. P., JÃ¸rgensen, F., Chhabra, S. R., et al. (1998). Construction and analysis of luxCDABE -based plasmid sensors for investigating N -acyl homoserine lactone-mediated quorum sensing. *FEMS Microbiol. Lett.* 163, 185–192. doi:10.1111/j.1574-6968.1998.tb13044.x.

5. Generous gift from the *Caenorhabditis elegans* Center at University of Minnesota, Minneapolis, MN.

6. Heurlier, K., Denervaud, V., Haenni, M., Guy, L., Krishnapillai, V., and Haas, D. (2005). Quorum-Sensing-Negative (*lasrlasr*) Mutants of Pseudomonas aeruginosa Avoid Cell Lysis and Death. *J. Bacteriol.* 187, 4875–4883. doi:10.1128/JB.187.14.4875-4883.2005.

Supplementary Methods:

Enzyme activity

Reactions were set up in triplicate in a 96 well-plate including 180 µl of Phosphotriesterase (PTE) buffer (50mM HEPES, 150mM NaCl, .2mM CoCl2), 10 µl enzyme (Ssopox W263I or GcL) at 1 mg/ml, 10 µl ethyl paraoxon (Millipore-Sigma, Burlington, MA) at a final concentration of 20 mM dissolved in methanol, or blank (PTE buffer and ethyl paraoxon).

The production of p-nitrophenolate was measured at 412 nm per well using a Synergy HTX plate reader (BioTek, USA). The Michaelis-Menten equation was used to determine enzyme activity. The experiment was performed in triplicate, with enzyme in LB media at 0 hours, and after 20 hours of incubation at 37˚ C, 250 rpm.
